# Spontaneous Complete Remission in a Patient with Acute Myeloid Leukemia and Severe Sepsis

**DOI:** 10.1155/2017/9593750

**Published:** 2017-07-24

**Authors:** Rambod Mozafari, Mahsa Moeinian, Ali Asadollahi-Amin

**Affiliations:** ^1^Department of Hematology, Imam Khomeini Hospital, Tehran University of Medical Sciences, Tehran, Iran; ^2^Research Center for Rational Use of Drugs, Tehran University of Medical Sciences, Tehran, Iran; ^3^Department of Infectious Disease, Imam Khomeini Hospital, Tehran University of Medical Sciences, Tehran, Iran

## Abstract

Without treatment, acute myeloid leukemia (AML) is almost always fatal. Spontaneous remission of AML is a rare phenomenon and usually with a short duration. The exact mechanisms are unknown. However, its association with infection and blood transfusions has been described. We report a 53-year-old male who presented with severe sepsis and who was diagnosed with AML (M4). He has experienced complete spontaneous remission with relatively long duration. To the best of our knowledge, it is the first case of spontaneous remission described in Iran.

## 1. Introduction

Acute myeloid leukemia (AML) manifests by proliferative, undifferentiated hematopoietic cells which infiltrate to blood, bone marrow, and other tissues. Approximately 10% of patient present with fever. Following chemotherapy, complete remission (CR) was defined with blood neutrophil and platelet count ≥ 1000/*µ*L and ≥100000/*µ*L, respectively, with no circulating blasts and blasts in bone marrow <5% [1]. Spontaneous remission (SR) without chemotherapy, complete or partial, is a very rare event with usually short duration [2]. It was first described following typhoid infection by Eisenlohr in 1878 [3, 4].

Until 2014, according to modern criteria, only 46 eligible cases were found [5]. Several events like infection and blood transfusion are suggested to play an important role in SR. However, the exact mechanism of this phenomenon has yet to be determined [6]. One hypothesis is immune activation by these events has antileukemic effects [7]. Recent progression in early diagnosis and treatment makes SR infrequent [8].

We report a case of AML without cytogenetic abnormalities who had spontaneous complete remission after severe sepsis and both blood and platelet transfusion without chemotherapy.

## 2. Case Report

A 53-year-old male presented with severe dyspnea, chest pain, productive cough, and dizziness. On admission, he was confused and pale with high-grade fever (39.5°C), tachypnea, and tachycardia, and blood pressure was 80/60 mmHg. There was no gingival hyperplasia and no peripheral lymphadenopathy or organomegaly. Chest X-ray revealed bilateral infiltrative abnormalities. He was transferred to Intensive care units (ICU) due to respiratory distress and mechanical ventilation started.

**Figure 1:**
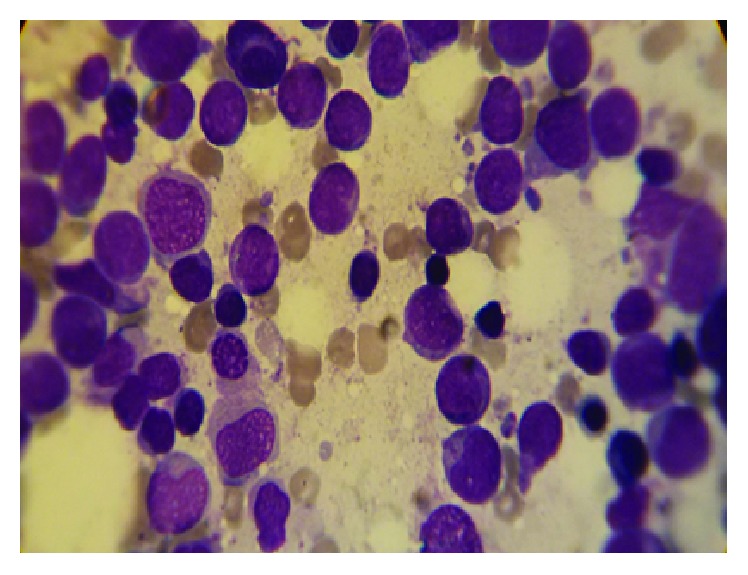
Bone marrow smear at diagnosis showed 70%–80% cellularity with decreased megakaryocyte, increased proportion of myeloid to erythroid cells, and high level of myeloid blasts which could be presumptive of AML-M4.

**Figure 2:**
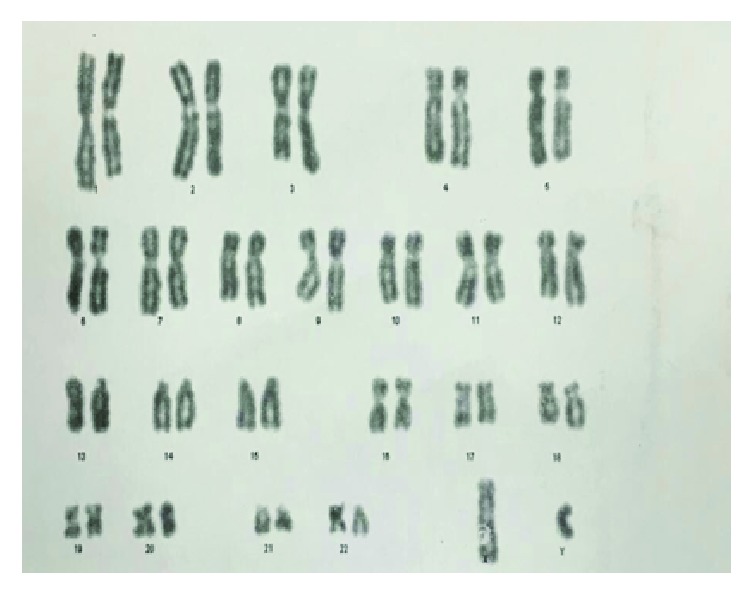
Karyotype analyses showed 46 XY compatible with the apparently normal male with no chromosomal aberration on the basis of GTG technique at 350–400 band resolution.

**Figure 3:**
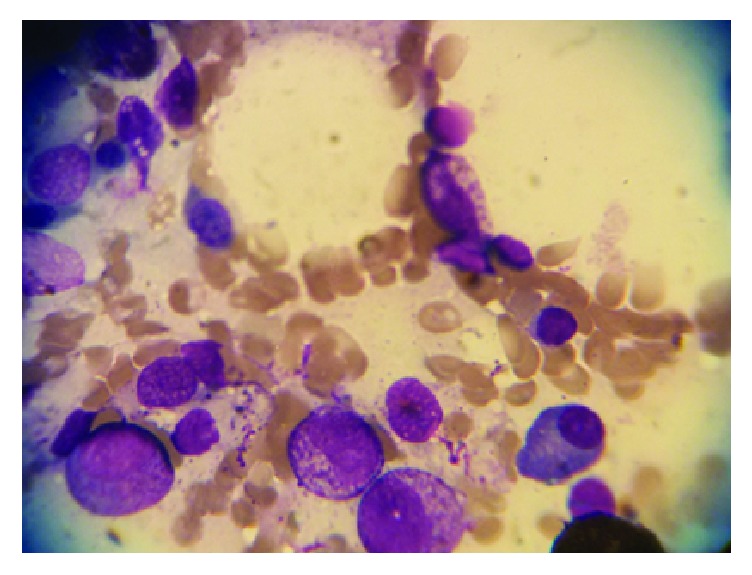
Bone marrow smears at spontaneous remission (2 weeks after sepsis resolution) showed 5–10% cellularity including sufficient megakaryocytes, myeloid and elytroid cells being in various maturation phases, and no blast.

**Figure 4:**
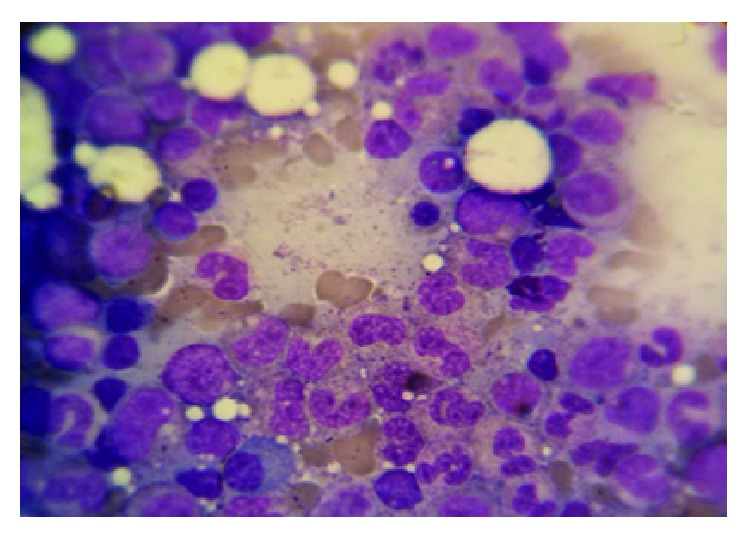
Bone marrow smear at spontaneous remission (4 weeks after sepsis resolution) showed 50–60% cellularity including sufficient megakaryocytes, the normal ratio of myeloid to elytroid cells, and no blast.

On admission, Laboratory findings were as follows: white blood cell (WBC), 400/*μ*L, hemoglobin (Hgb), 6 g/dl, platelet (PLT) 54000/*μ*L, C-reactive protein (CRP), 33 mg/L, and erythrocyte sedimentation rate (ESR), 125 mm/h. All of the renal and liver function tests, coagulation test, and electrolytes were within the normal values. The bone marrow aspiration smear (Figure 1) revealed 70%–80% cellularity with reduced megakaryocytes and raised ratio of myeloid to erythroid cells. Following bone marrow biopsy, the immunophenotyping analysis showed more than 80% blasts with positive CD13, CD14, CD33, CD34, CD45, CD64, HLA-DR, and c-MPO supported the AML-M4 based on French-American-British classification. Cytogenetic analysis demonstrated 46 XY compatible with the apparently normal male without chromosomal abnormality (Figure 2). The chemotherapy was postponed due to severe infection. The culture of tracheal discharge was positive for* Enterobacter* spp. and* Acinetobacter baumannii*. Blood culture result was* Klebsiella* pneumonia. Broad-spectrum antibiotics were started, and antifungal drug was added to antibiotics owing to persistent fever and neutropenia. The patient received red cells (10 units), platelet transfusions (10 units), and low-dose corticosteroid (dexamethasone 4 mg every 12 hr) during admission. Two weeks later, the infections completely resolved and he was weaned off the ventilator. Two weeks after sepsis resolution, hematologic status improved markedly with WBC of 3880/*μ*L, Hgb of 9.2 g/dl, and PLT of 203000/*μ*L. In addition, the bone marrow smear contained 5–10% cellularity with adequate megakaryocytes for this setting, myeloid and elytroid cells being in various maturation phases, and no blasts were recognized (Figure 3). Two weeks later, the bone marrow biopsy was repeated and the smear revealed 50–60% cellularity with sufficient megakaryocyte, the normal ratio of myeloid to erythroid cells, and no blast (Figure 4). The concurrent complete blood count (CBC) revealed WBC, 14600/*μ*L, with neutrophil, 72.3%, Hgb, 9.4 g/dl, and PLT, 392000/*μ*L (i.e., spontaneous complete remission). The immunophenotyping analysis showed normal plasma cell population. After 18 months, he has been in remission phase with WBC, 4800/*μ*L, neutrophil, 69.0%, Hgb, 12.3 g/dl, and PLT, 253000/*μ*L.

## 3. Discussion

According to the CR definition, our patient is in SCR until this manuscript is written (18 months). Among 46 eligible patients reported by Rashidi and Fisher as cases of SR, 39 patients achieved SCR with the median duration of remission only 5 months, whereas only nine patients had durable CR (defined as CR lasting for 1 year or longer). Therefore, our patient could be added to the latter group (Table 1) [9]. In other reports, the median period of remission was 8.2 ± 9.8 months [10].

Infections and blood transfusion lead to immune activation and have been frequently reported with the majority of SR cases like our case. However, exact mechanisms are not well known.

Moreover, cases unrelated to transfusions or infections have been documented previously. As in our case, pneumonia and bacteremia were significantly more common among CR cases compared to partial remission [5].

Hypergammaglobulinemia due to activation of cytotoxic T-cell lymphocyte, which recognizes blasts, or against infectious antigens with cross-reactivity to leukemic blasts increased levels of interleukin-2 (IL-2) which activate natural killer cells (NK), tumor necrosis factor-*α* (TNF-*α*), interferon-*γ*, IL-1, IL-6, heat shock proteins (HSPs), and ultimately leukocytoclastic vasculitis, all have been proposed to play a role for SR during immune activation [9, 11]. Furthermore, infection induces the production of granulocyte colony-stimulating factor (G-CSF) which can increase the effector cytotoxic cells and suppression of leukemic cells regeneration [10].

Moreover, immunotherapy with bacterial extracts like streptococcus pyogenes, vaccines such as Calmette–Guérin (BCG), endotoxins, use of bispecific T-cell engager (BiTE) antibodies, and T-cells expressing chimeric antigen receptors (CARs) were associated with a high rate of remission and restricted effect on survival time in AML [4, 9, 12].

Nonirradiated blood transfusion, which entailed the donor immune factors like cytokines, NK cell, and T-cell, creates a condition like graft-versus-leukemia/lymphoma in allogeneic bone marrow transplant against malignant leukemic cells. In addition, antileukemic antibodies can play a role. Thus, routine use of leukocyte depleted blood products which were irradiated in patients with AML may reduce the chance of transfusions related SR [5, 13].

Finally, although our patient did not receive a high dose of methylprednisolone like Shimohakamada report, inconsistent with other reports, unspecified doses of corticosteroids may have a role [6, 8].

It seems the SCR in our case resulted from both septic condition and nonirradiated blood and platelet transfusion. Most SR cases had short duration [9], so postremission (consolidation) therapy or bone marrow transplant (BMT) as indicated is critical [1]. Therefore, further investigations are required to determine whether further therapy should be started in these situations or not.

## Figures and Tables

**Table 1:** Compare the characteristics of this case with previous similar cases reported by Rashidi and Fisher.

Characteristics	Age	Sex	FAB	Karyotype	BM blasts (%) at presentation	Infection	Type	Fever	Irradiated transfusion	Remission	Duration	Relapse
This case	53	M	M4	Normal	>80%	+	Pneumonia	+	+	CR	18 m	—
Number of previously similar cases	5	27	5	9	11	32	13	37	32	39	3	8

FAB: French-American-British classification; BM: bone marrow; CR: complete remission; m: months; M: male.
